# THE ROLE OF CONTEXTUAL FACTORS IN ADULT DEVELOPMENT AND AGING

**DOI:** 10.1093/geroni/igad104.1580

**Published:** 2023-12-21

**Authors:** Gizem Hueluer, Takeshi Nakagawa, Hans-Werner Wahl

## Abstract

Individual development is embedded in multiple environmental systems, ranging from broader distal cultural and historical contexts to more proximal residential, family, and partnership contexts. The goal of this symposium is to showcase research studying the role of distal and proximal contextual factors in the development and aging of key domains, including health, cognitive function, social relationships, and emotional experiences. Nakagawa et al. examine cultural differences in the associations of positive affect and loneliness with longevity using data from prospective surveys in the United States (1986–2005) and Japan (1987–2006). Hülür et al. use 35-year longitudinal data (1977-2012) from 3,482 participants of the Seattle Longitudinal Study to examine associations between perceived residential neighborhood characteristics and trajectories of cognitive aging. Kobayashi et al. study cohort and gender differences in trajectories of social contact and social participation and the effects of being married and living with a child using data from the National Survey of the Japanese Elderly (1987-2021). Meier et al. will present data from a study on emotional co-regulation in younger and older couples’ daily lives focusing on situational factors (e.g., psychological availability) within romantic relationships that can shape daily emotional experiences and relationship quality. The discussion by Hans-Werner Wahl will focus on how these approaches contribute to our understanding of processes of context dynamics and how they can be utilized to promote the maintenance of health and function in aging.

